# Changes in the bioactive properties of strawberries caused by the storage in oxygen‐ and carbon dioxide‐enriched atmospheres

**DOI:** 10.1002/fsn3.1099

**Published:** 2019-07-15

**Authors:** Franco Van de Velde, Debora Esposito, John Overall, María Paula Méndez‐Galarraga, Mary Grace, María Élida Pirovani, Mary Ann Lila

**Affiliations:** ^1^ Facultad de Ingeniería Química, Instituto de Tecnología de Alimentos Universidad Nacional del Litoral Santa Fe Argentina; ^2^ Consejo Nacional de Investigaciones Científicas y Técnicas (CONICET) Santa Fe Argentina; ^3^ Food Bioprocessing and Nutrition Sciences Department, Plants for Human Health Institute North Carolina State University Kannapolis North Carolina; ^4^ Department of Animal Science North Carolina State University Raleigh North Carolina

**Keywords:** 2,2‐diphenyl‐1‐picrylhydrazil, cyclooxygenase‐2, ferric reducing antioxidant power, human dermal fibroblast migration, interleukin‐6, modified atmosphere storage

## Abstract

The changes in the antioxidant capacity, anti‐inflammatory, and wound healing properties of strawberry fruits as a consequence of the storage in atmospheres enriched in oxygen and carbon dioxide were investigated. Berries were exposed to two different gas compositions: 70% O_2_ + 20% CO_2_ and 90% O_2_ + 10% CO_2_, and stored for up to 20 days at 5°C. The antioxidant capacity, assessed through DPPH and FRAP methods, decreased around 17% in samples exposed to 70% O_2_ + 20% CO_2_ at day 20. However, the antioxidant activity of fruits stored in 90% O_2_ + 10% CO_2_ was maintained until day 20 and experienced an increase of around 10% on day 10. Moreover, strawberry stored in 90% O_2_ + 10% CO_2 _at days 5–10 showed an improved suppression of the pro‐inflammatory genes Cox‐2 and iNOS up to 30% higher than samples at day 0 in an in vitro LPS‐stimulated RAW 264.7 macrophage culture. In addition, berries exposed to 90% O_2_ + 10% CO_2_ at day 10 showed a human dermal fibroblast migration 30% higher than samples at day 0 in an in vitro skin‐fibroblast‐migration model. Therefore, evidence suggests that strawberry storage in 90% O_2_ + 10% CO_2_ can be a promissory alternative to offer fruits with enhanced bioactivity.

## 1 INTRODUCTION

Strawberry is an extraordinary fruit appreciated for its typical aroma, bright red color, and sweetness, and it is considered an important source of nutraceutical compounds such as vitamin C and polyphenolic compounds (da Silva Pinto, Lajolo, & Genovese, 2008; Van de Velde, Tarola, Güemes, & Pirovani, 2013; Nowicka, Kucharska, Sokół‐Łętowska, & Fecka, 2019). Anthocyanins, ellagitannins, proanthocyanidins, and some other flavonoids are important phenolic compounds in strawberry, which besides being responsible for certain sensorial characteristics, they impart relevant biological properties to the consumers such as antioxidant, anti‐inflammatory, anti‐atherosclerotic, and anticarcinogenic (Crecente‐Campo, Nunes‐Damaceno, Romero‐Rodríguez, & Vázquez‐Odériz, 2012; Giampieri et al., 2017; Nowicka et al., 2019).

Several chronic pathologies such as type 2 diabetes mellitus, cardiovascular diseases, among others, are induced by a persistent pro‐inflammatory status; thus, the dietary strategies that can reduce the inflammatory status are valued (Joseph, Edirisinghe, & Burton‐Freeman, 2014). The anti‐inflammatory effects of strawberry crude extracts or purified fractions were recently described (Giampieri et al., 2017; Joseph et al., 2014), and an anti‐inflammatory mechanism principally associated with strawberry main anthocyanin pelargonidin‐3‐*O*‐glucoside was proposed (Duarte et al., 2018).

Moreover, bioactive compounds from strawberry also might have some positive effects on the wound healing process. Wound healing is a complicated physiological process that includes several phases including the inflammation phase, a proliferation phase, and a remodeling phase (Lindley, Stojadinovic, Pastar, & Tomic‐Canic, 2016). In the proliferative phase, lots of cells such as fibroblasts, keratinocytes, and endothelial cells are recruited in the injury area with the aim to close the wound (Lindley et al., 2016). In that sense, an anthocyanin‐enriched fraction prepared from strawberries showed an improved migration of human dermal fibroblasts in *an vitro* study (Van de Velde, Esposito, Grace, María, & Lila, 2018). Hence, polyphenolic compounds from strawberries may offer and interesting and natural alternative as wound healing agents.

Although the intake of strawberries is being promoted due to health benefits, the shelf life of fruit is extremely short as a result of the high perishability given by mechanical damage susceptibility, microbial decay, water loss, and physiological senescence (Zheng et al., 2007). The application of cold through precooling and refrigerated storage are the most used strategies to preserve the product (Nalbandi, Rangbar, Ghasemzadeh, & Seiiedlou, 2016). However, these operations do not completely eliminate changes that occur during storage of the product, so strawberry environment is generally complemented with modified atmospheres containing 15%–20% carbon dioxide (Odriozola‐Serrano, Soliva‐Fortuny, & Martín‐Belloso, 2010; Pelayo, Ebeler, & Kader, 2003; Pérez & Sanz, 2001). In addition, it is been proposed that the use of elevated O_2_ atmospheres (>21%) in combination with high CO_2 _concentrations may extend the storage life of strawberries longer (Allende, Marín, Buendía, Tomás‐Barberán, & Gil, 2007; Zhang, Samapundo, Pothakos, Sürengil, & Devlieghere, 2013; Van de Velde et al., 2019). Moreover, added to the beneficial effects that can be achieved on the shelf life of fruit, it was postulated that the storage of strawberries in O_2 _concentrations higher than 60% promotes the synthesis of polyphenolic compounds due to a physiological response to stress, via the activation of the phenylpropanoid metabolism (Cisneros‐Zevallos, 2006; Zheng et al., 2007). Therefore, this technology can be used as an excellent opportunity to offer strawberries with an extended shelf life along with an extra content of phenolic compounds which may exhibit an improved bioactivity.

The storage of strawberries at 5°C for 20 days in 70% O_2_ + 20% CO_2_ and 90% O_2_ + 10% CO_2_ effectively controlled the microbial decay and minimally affected the general quality attributes of fruits. Moreover, treatments also caused an important accumulation of phenolic compounds such as anthocyanins, flavonols, phenolic acids, and ellagitannins up to 167% at specific days of the storage (Van de Velde et al., 2019). However, strawberry vitamin C content was reduced up to 50% during storage due to the pro‐oxidant environment inside containers. Hence, based on the evidence that the storage of strawberries in O_2_‐ and CO_2_‐enriched atmospheres can elicit positive and negative effects on the phytochemical content of fruits, the impact of these treatments on the bioactive properties offered by berries needs to be clarified.

Thus, the objective of this work was to determine the effects of the storage of strawberries in two O_2_‐ and CO_2_‐enriched atmospheres (70% O_2_ + 20% CO_2_ and 90% O_2_ + 10% CO_2_) on the antioxidant capacity, the anti‐inflammatory, and the wound healing properties of the fruits.

## 2 MATERIALS AND METHODS

### 2.1 Plant material

Strawberries (*Fragaria x ananassa* Duch.) cv. “San Andreas” were harvested from one field at Coronda (31°58′00″S 60°55′00″W), Santa Fe, Argentina, during 2017. Fruits were transported to the laboratory of the Instituto de Tecnología de Alimentos, Universidad Nacional del Litoral, Argentina, and stored under refrigeration until use.

### 2.2 Sample preparation

Sample preparation and atmosphere storage treatments were described in detail by Van de Velde et al. (2019). Briefly, conditioned strawberries were placed in hermetic jars with lids (~300g per jar, ~ 15 fruits per jar). Two gas combinations were prepared: 70% O_2_ + 20% CO_2_, balanced with N_2_, and 90% O_2_ + 10% CO_2_. Four jars were prepared per each atmosphere combination and stored at 5°C for 20 days. Gas composition was digitally controlled and recalibrated to target values in each jar every day. At days 0, 5, 10, and 20, jars were taken out one at a time from the refrigerated unit, and then, strawberries were removed from containers, and frozen at −80°C until lyophilization in a Flexy‐dry freeze dryer (SP Scientific, NY). Analyses were performed on the extracts prepared with the freeze‐dried materials as explained below.

### 2.3 Preparation of extracts

Extraction procedures for antioxidant capacity analysis and for cell culture experiments from lyophilized strawberries were described by Van de Velde et al. (2018).

### 2.4 Antioxidant capacity analyses

#### 2.4.1 Free radical scavenging (DPPH) assay

The antioxidant capacity was estimated by determining the ability of samples to scavenge the free radical 2,2‐diphenyl‐1‐picrylhydrazil (DPPH) according to Teow et al. (2007). The determinations were made in triplicate, and results were calculated using a standard curve of (±)‐6‐Hydroxy‐2,5,7,8‐tetramethylchromane‐2‐carboxylic acid < Trolox> (0.1 – 0.5 mM) and expressed as mmol Trolox Kg^‐1^ of fresh weight.

#### 2.4.2 Ferric reducing antioxidant power (FRAP) assay

The antioxidant capacity was also conducted according to Benzie and Strain (1996), monitoring the absorbance change at 593 nm caused by the reduction of the Fe^3+^‐2,4,6‐tris(2‐pyridil)‐s‐triazine (TPTZ) complex to the ferrous form at pH 3.6. FRAP values were obtained by comparing the absorbance change in the samples with those obtained from increasing concentrations of Fe^2+ ^(500 – 6,000 µmol FeSO_4_ L^‐1^). Results were expressed as mmol Fe^2+^ Kg^‐1^ of fresh weight.

### 2.5 Anti‐inflammatory and wound healing properties

#### 2.5.1 Cell lines

The mouse macrophage cell line RAW 264.7 (ATCC TIB‐71, obtained from American Type Culture Collection, Livingstone, MT, USA) and the primary human dermal fibroblasts isolated from adult skin (HDFa, Invitrogen C‐013‐5C) were used for studying the anti‐inflammatory and wound healing properties of the extracts, respectively. Culture conditions and cell requirements were described in detail by Van de Velde et al. (2018).

#### 2.5.2 Cell viability and dose range determination

The cytotoxicity of samples against mouse macrophages and human fibroblasts was evaluated spectrophotometrically after 24 hr of exposure of cells to extracts in a concentration range from 50 to 250 mg/L (dry weight volume^‐1^) and the addition of MTT ([3‐(4, 5‐dimethylthiazol‐2‐yl)‐2, 5‐diphenyl‐tetrazolium bromide], as early described (Mosmann, 1983).

#### 2.5.3 Reactive oxygen species (ROS) assay

The fluorescent dye dichlorodihydrofluorescein diacetate acetyl ester (H_2_DCFDA) was used for determining the production of reactive oxygen species (ROS) in the lipopolysaccharide (LPS)‐stimulated macrophages (LPS from Escherichia coli 0,127:B8, final concentration: 1 mg/L) and to analyze the changes after sample incorporation, according to Choi, Hwang, Ko, Park, and Kim (2007).

#### 2.5.4 Nitric oxide (NO) assay

The ability of test samples to suppress the NO radical formation in LPS‐activated macrophages was determined as described by Kellogg and Lila (2013). Dexamethasone (Dex) at 10 μM was used as a positive control. Results were expressed as µmol NO L^−1^.

#### 2.5.5 Anti‐inflammatory in vitro assay

The anti‐inflammatory activity of samples was assessed by treating LPS‐stimulated macrophages with sample extracts followed by RNA extraction, purification, and cDNA synthesis and quantitative PCR analysis as described by Van de Velde et al. (2018). Inducible oxide nitric synthase (iNOS), cyclooxygenase‐2 (Cox‐2), interleukin‐6 (IL‐6), and interleukin 1β (IL‐1*β*) were the gene primers studied. Dex at 10 μM was used as a positive control.

### 2.6 In vitro skin fibroblast migration and proliferation assay

The analysis was conducted according to Van dee Velde et al. (2018). Briefly, HDFa fibroblasts were seeded into 96‐well Oris™ plate (Platypus Technologies, LLC) at a concentration of 3 × 10^5^ cells/ml and cultured to nearly confluent cell monolayers. Cells were labeled with NucBlue® Live Cell Stain and CellTracker™ Red CMTPX (at 1 µM) fluorescent dyes. After confluence was reached, the well inserts were removed to form an unseeded region (2 mm in diameter) at the center of each well. Then, growth medium was removed and free cellular debris was removed by washing cells once with sterile phosphate‐buffered saline (PBS). Fresh growth medium containing vehicle (0.8% ethanol), positive control (0.5% fetal bovine serum, FBS), or extracts (final concentrations: 50 mg/L) was added to a set of 4 wells per dose and incubated up to 48 hr at 37°C with 5% CO_2_. The progress of cell migration was materialized by the cell movement into the unseeded region located at the center of each well and was monitored 0, 24, and 48 hr after sample addition by measuring the fluorescence at excitation/emission wavelength of 360/460 nm and 577/605 nm on a microplate reader (Synergy H1, Biotech). Bright field and fluorescent images were observed using EVOS® FL Auto Cell Imaging System (Life Technologies). Images were captured as at center of well for consistency purposes. Three representative images of the areas from each well under each condition were photographed at 0 and 48 hr to estimate the wound closure.

### 2.7 Statistical analysis

Statistics were performed by means of the software GraphPad Prism v6 (GraphPad Software Inc.). All data were analyzed by one‐way ANOVA. Significant differences among means were determined by Tukey's test at 5% level of significance. All results are expressed as means ± standard deviation (*SD*).

## 3 RESULTS AND DISCUSSION

### 3.1 Effects of refrigerated storage of strawberries in O_2_‐ and CO_2_‐enriched atmospheres on the antioxidant capacity

**Figure 1:**
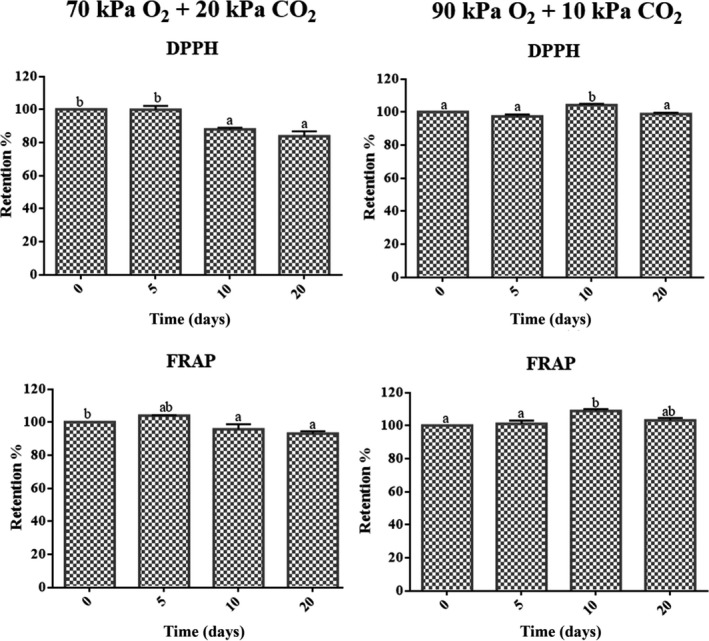
Effects of strawberry storage in 70% O_2_ + 20% CO_2 _and 90% O_2_ + 10% CO_2_ on antioxidant capacity analyzed by the DPPH and FRAP methods. Changes are reported as the mean ± *SD*. Means not marked by the same letter are significantly different (*p* ≤ 0.05) according to Tukey's comparison test

Figure 1 shows changes in the in vitro antioxidant capacity of “San Andreas” strawberries measured through the DPPH and FRAP methods. The antioxidant activity of fruits at day zero was 9.6 ± 0.5 mmol Trolox Kg^‐1^ and 0.48 ± 0.03 mmol Fe^2+^ Kg^‐1^, according to the DPPH and FRAP assays, respectively. Results were in the range of results reported by both methodologies in “San Andreas” strawberries (Fernandes, Domingues, Freitas, Delerue‐matos, & Mateus, 2012) and other strawberry varieties (Nowicka et al., 2019).

The antioxidant capacity by DPPH of strawberries stored in 70% O_2_ + 20% CO_2_ was maintained during the first 5 days and then diminished approximately 17% by the end of storage (day 20). The antioxidant ability of samples, measured by FRAP methodology, showed the same trend, but only diminished by 7% at the end of storage (Figure 1).

The changes in the antioxidant capacity assessed by DPPH of strawberries stored in 90% O_2_ + 10% CO_2_ showed a different behavior, in that initially the values were stable, then increased 5% by day 10, and finally returned to prestorage values by day 20. In the same way, the antioxidant capacity of these samples as measured by FRAP was maintained during the first 5 days of storage, then experienced an increase (10%) at day 10, and finally returned to the initial value at day 20 (Figure 1).

Odriozola‐Serrano et al. (2010) reported that the antioxidant capacity through the ABTS method of strawberry wedges stored for 21 days under high O_2_ atmospheres (60 and 80% O_2_) markedly increased (up to 25%) after day 4 of storage and finally decreased beyond day 9 up to 82% of the initial value. In addition, Zheng et al. (2007) reported that the antioxidant activity of strawberries stored in atmospheres with high O_2 _concentrations, ranging from 40% to 100%, increased 1.2‐fold after 7 days of storage.

The changes in the antioxidant capacity of strawberries stored under O_2_‐ and CO_2_‐enriched atmospheres may be attributable to the changes in the amount of phytochemical compounds during storage. About that, the levels of vitamin C, proanthocyanidins, and flavonols of strawberries stored in 70% O_2_ + 20% CO_2_ remained almost unchanged throughout the storage period in a previous study (Van de Velde et al., 2019). However, the retention of anthocyanins in these samples remained constant until day 10 of storage, but after that time, anthocyanins were degraded to different degrees (Van de Velde et al., 2019). Hence, the decrease in the antioxidant capacity observed for strawberries exposed to 70% O_2_ + 20% CO_2_ can be principally due to the decrease in the anthocyanins experienced by these samples.

On the contrary, as previously determined, the anthocyanin concentrations of strawberries exposed to 90% O_2_ + 10% CO_2_ substantially increased (up to 167%) during the first 5 days of storage. Thereafter, anthocyanin content decreased gradually and remained even higher than their initial values at the end of storage (Van de Velde et al., 2019). This behavior was associated with a physiological response to stress caused by the altered gas composition on the strawberry tissues. However, this phenomenon was not seen for berries stored at 70% O_2_ + 20% CO_2_. Therefore, the increase in the antioxidant capacity registered by strawberries exposed to 90% O_2_ + 10% CO_2_ at day 10 may be the result of the increase in the anthocyanins experimented during the storage for these samples. However, the retention of vitamin C in strawberries treated with 90% O_2_ + 10% CO_2_ progressively decreased and reached 50% of the initial content at day 20 (Van de Velde et al., 2019). As ascorbic acid and polyphenolic compounds contribute significantly to the antioxidant activity of strawberries (Van de Velde et al., 2013), it might be inferred that the vitamin C degradation previously perceived for strawberries stored under this treatment could have curtailed the magnitude of the antioxidant capacity increment, which was not higher than 10% at day 10 (Figure 1).

### 3.2 Effects of refrigerated storage of strawberries in O_2_‐ and CO_2_‐enriched atmospheres on anti‐inflammatory properties

#### 3.2.1 Effects of strawberry extracts on cell viability

Strawberry crude extracts prepared in 80% ethanol in the range of 50 – 250 mg/L did not depress cell viability (*p* > 0.05) (data not shown). Therefore, all following experiments were carried out at 50 mg/L. Any inhibitory effect of the extracts at this concentration will be attributable to biological effects and not to cytotoxic effects.

#### 3.2.2 Changes on intracellular reactive oxygen species (ROS) and nitric oxide (NO) production

**Figure 2:**
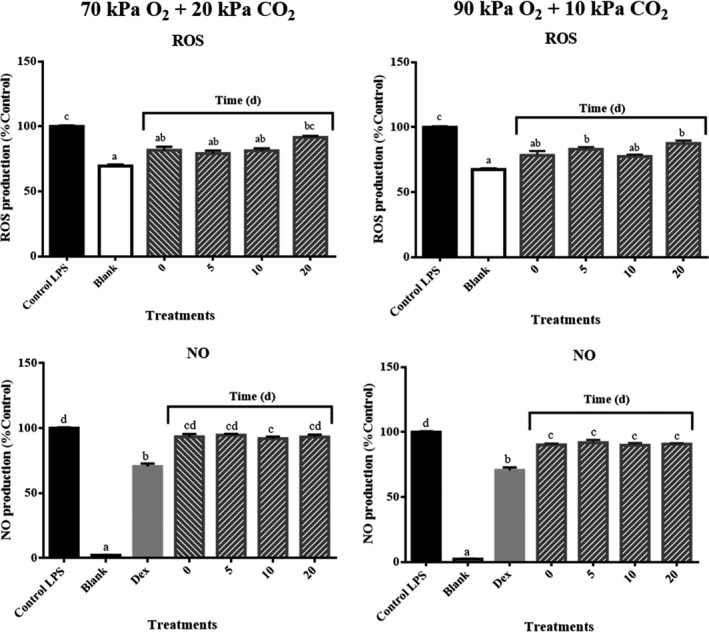
Effects of strawberry storage in 70% O_2_ + 20% CO_2_ and 90% O_2_ + 10% CO_2_ on reactive oxygen species (ROS) production and on nitric oxide (NO) production. LPS final concentration: 1 mg/L. Strawberry sample final concentrations: 50 mg/L. Dex at 10 μM was used as positive control for NO analysis. Changes reported as the mean ± *SD* relative to LPS controls. Means not marked by the same letter are significantly different (*p* ≤ 0.05) according to Tukey's comparison test

“San Andreas” strawberry crude extracts at day zero exhibited a 20% reduction in ROS production compared to cells treated only with LPS (*p* ≤ 0.05) (Figure 2). This result was in agreement with Gasparrini et al. (2017), who reported a reduction close to 35% in the amount of ROS in activated macrophages treated with “Alba” strawberry crude extract at 100 mg/L. The ability of extracts from strawberries stored in 70% O_2_ + 20% CO_2_ to reduce ROS production in the LPS‐activated macrophages was maintained until day 10. At the end of storage (day 20), these fruits reduced the ROS production compared to LPS control but not significantly (Figure 2). However, samples exposed to 90% O_2_ + 10% CO_2_ maintained the decrease in ROS production until the end of storage (day 20) (Figure 2).

The mechanism by which phytochemicals from strawberries reduce ROS production in LPS‐stimulated RAW 264.7 macrophages was suggested to be as a result of the increase in endogenous antioxidant enzyme activities, which includes glutathione reductase, glutathione transferase, and superoxide dismutase (Gasparrini et al., 2017).

In addition, previous results suggest that proanthocyanidins purified from strawberry may be the active polyphenols against the oxidative stress in LPS‐stimulated macrophages (Van de Velde et al., 2018). As determined in preceding results, the retentions of proanthocyanidins were not affected or slightly diminished in strawberries stored in the two high oxygen and high carbon dioxide atmospheres studied for up to 20 days (Van de Velde et al., 2019). Therefore, the exposure of strawberries to these altered gas composition atmospheres did not affect their ability to reduce ROS throughout storage, most likely because proanthocyanidins were not degraded.

On the other hand, as shown in Figure 2, strawberry extracts of samples exposed to 90% O_2_ + 10% CO_2_ slightly suppressed NO synthesis (<10%) at day zero, and this level of inhibition was maintained throughout the storage period. Strawberries stored in 70% O_2_ + 20% CO_2_ showed this statistically significant (*p* ≤ 0.05) slight suppression in the NO synthesis (<10%) but only in samples at day 10 (Figure 2). The NO synthesis suppression can be explained, as observed for ROS suppression, due to proanthocyanidins were responsible for the NO synthesis reduction (Van de Velde et al., 2018), and as a consequence that these compounds were not affected by the studied gas environments (Van de Velde et al., 2019).

#### 3.2.3 Changes in inflammatory markers

**Figure 3:**
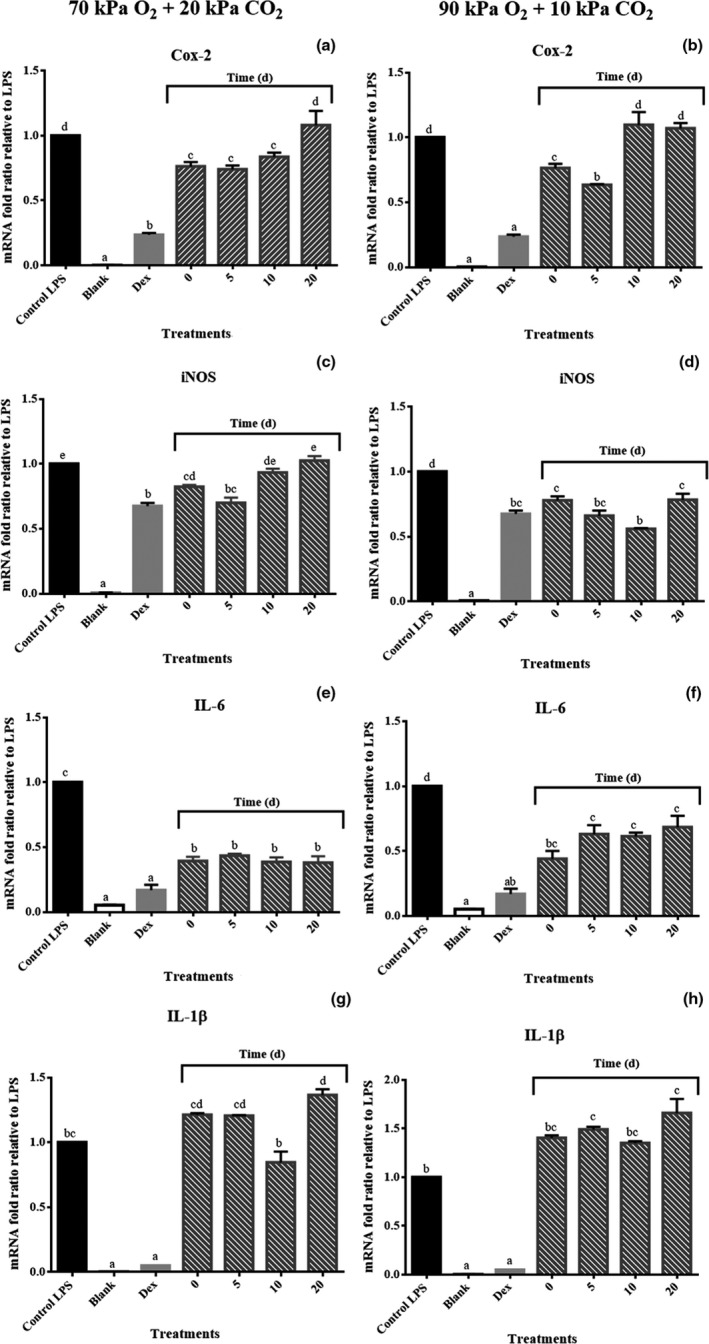
Effects of strawberry storage in 70% O_2_ + 20% CO_2 _and 90% O_2_ + 10% CO_2 _on pro‐inflammatory gene expression profile: (a and b) cyclooxygenase‐2 (Cox‐2), (c and d) iNOS, (e and f) cytokine interleukin‐6 (IL‐6), and (g and h) cytokine interleukin‐1β (IL‐1β). LPS final concentration: 1 mg/L. Strawberry sample final concentrations: 50 mg/L. Dex at 10 μM used as positive control. Fold changes in gene expression reported as the mean ± *SD* relative to LPS controls. Means not marked by the same letter are significantly different (*p* ≤ 0.05) according to Tukey's comparison test

“San Andreas” strawberry crude extracts at day zero significantly (*p* ≤ 0.05) suppressed Cox‐2 gene expression based on 25% change relative to macrophages treated only with LPS (Figure 3a and b). The suppression of Cox‐2 was maintained until day 10 of storage in samples exposed to 70% O_2_ + 20% CO_2_. Thereafter, gene suppression decreased and no suppression was evidenced at day 20 (Figure 3a). However, samples exposed to 90% O_2_ + 10% CO_2_ increased Cox‐2 gene suppression 10% at day 5 in relation to samples at day 0, but no suppression was demonstrated during later days of storage (Figure 3b).

The expression of the iNOS gene, responsible for the secretion of NO, was suppressed nearly 20% by the strawberry extracts at day zero (Figure 3c and d). Extracts of samples stored in 70% O_2_ + 20% CO_2_ maintained gene inhibition capacity until day 5. Nevertheless, after that time, these berries were progressively losing gene suppression capacity, which was not significant (*p* > 0.05) at day 20 (Figure 3c). On the contrary, iNOS gene suppression progressively increased during the storage of samples exposed to 90% O_2_ + 10% CO_2_, reaching a gene inhibition near to 50% at day 10 (30% higher than inhibition produced by samples at day 0). Then, a decrease in the gene suppression was observed at day 20, but in the range of the gene inhibition experimented by samples at day 0 (Figure 3d).

Strawberry extracts inhibited IL‐6 expression based on 60% change relative to LPS‐stimulated control at day zero (Figure 3e and f). Samples stored in the atmosphere 70% O_2_ + 20% CO_2_ maintained the expression suppression of this gene throughout the storage period (Figure 3e). Meanwhile, strawberries exposed to 90% O_2_ + 10% CO_2_ at day 5 inhibited gene expression 20% less than samples at day 0, and this value was maintained until day 20 (Figure 3f).

Finally, strawberry extracts did not show any inhibitory effect on IL1‐β gene expression at least at the treated concentration (50 mg/L) at day 0 (Figure 3g and h). Samples stored in both atmospheres also showed no effect on this biomarker of inflammation during storage (Figure 3g and h).

As demonstrated in this work, strawberry crude extracts at 50 mg/L suppressed the expression of the pro‐inflammatory genes Cox‐2, iNOS, and IL‐6. The storage of strawberries in 70% O_2_ + 20% CO_2_ generally maintained these anti‐inflammatory effects for up to 20 days at 5°C. Moreover, strawberries stored in 90% O_2_ + 10% CO_2 _between days 5 to 10 improved gene suppressions up to 30% higher than samples at day 0. However, the inhibition of the pro‐inflammatory genes exerted for all strawberry samples was generally lower than that observed for the positive control “Dex.” The mechanism by which Dex exerts its anti‐inflammatory action in LPS‐activated RAW 264.7 cells was elucidated through the inhibition of early pro‐inflammatory cytokine IL‐1β gene expression by a mechanism involving the blocking of LPS‐induced NF‐kB/Rel and AP‐1 activation (Jeon et al., 2000).

In agreement, the expression of anti‐inflammatory mediators and cytokines induced by LPS was reduced by strawberry crude extracts in other studies achieved on RAW macrophages (Gasparrini et al., 2017; Liu & Lin, 2013). However, evidences of how a postharvest treatment based on an altered gas composition can negatively impact on these inflammatory markers were not early described.

Other authors reported that the significant anti‐inflammatory effect of strawberry extracts is partially due to the presence of high amounts of the anthocyanin pelargonidin‐3‐*O*‐glucoside, and an anti‐inflammatory molecular mechanism involving the NF‐κB and MAPK pathways was proposed (Duarte et al., 2018).

Therefore, the stronger effect against the expression of inflammatory markers Cox‐2 and iNOS exerted by the extracts prepared from strawberries exposed to 90% O_2_ + 10% CO_2 _can be linked with the concomitant increase in the content of pelargonidin‐3‐*O*‐glucoside previously observed (Van de Velde et al., 2019).

### 3.3 Effects of refrigerated storage of strawberries in O_2_‐ and CO_2_‐enriched atmospheres on wound healing properties

#### 3.3.1 Effects of strawberry extracts on cell viability

Strawberry extracts in the range of 50 – 250 mg/L did not depress human dermal fibroblast viability (*p* > 0.05) (data not shown). Hence, subsequent experiments were performed at the lowest nontoxic concentration of 50 mg/L.

#### 3.3.2 Changes in skin fibroblast migration

A cell migration assay on fibroblasts was conducted to determine the effect of “San Andreas” strawberry crude extracts on wound healing and to study changes in the cell migration due to the storage of berries in atmospheres with enriched levels of oxygen and carbon dioxide.

**Figure 4:**
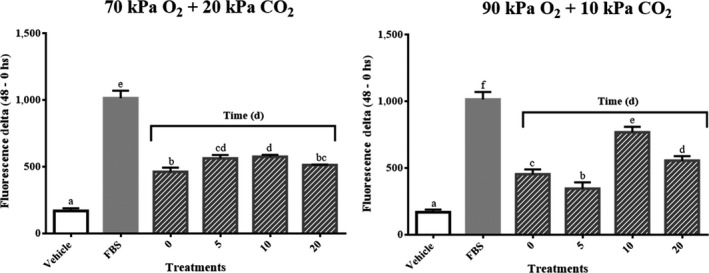
Effects of strawberry storage in 70% O_2_ + 20% CO_2 _and 90% O_2_ + 10% CO_2 _on skin fibroblast migration. Data reported as the mean ± *SD*. Strawberry sample final concentrations: 50 mg/L. FBS, fetal bovine serum at 0.5% used as positive control. Means not marked by the same letter are significantly different (*p* ≤ 0.05) according to Tukey's comparison test

**Figure 5:**
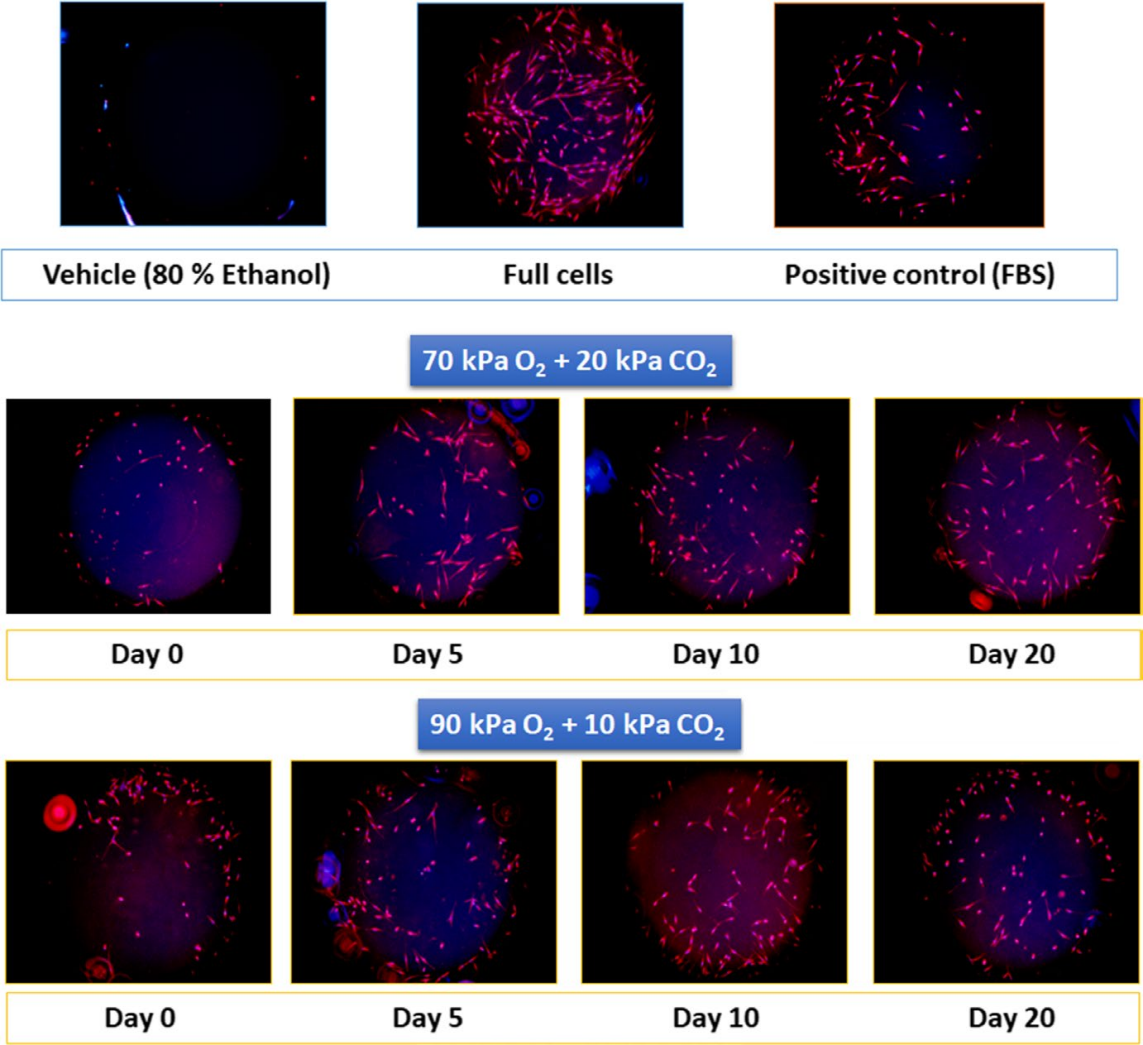
Progress of skin HDFa fibroblast migration after 48‐hr exposure with crude extracts of strawberries stored in 70% O_2_ + 20% CO_2 _and 90% O_2_ + 10% CO_2_. Bright field and fluorescent images were observed using EVOS® FL Auto Cell Imaging System (Life Technologies). FBS: fetal bovine serum at 0.5% used as positive control, Full cells correspond to wells seeded without stopper

Strawberry crude extracts at day zero enhanced cell migration after 48 hr in a percentage equal to 45% of the migration registered for the positive control FBS (Figure 4 and Figure 5). Samples exposed to 70% O_2_ + 20% CO_2_ slightly increased the cell migration around 10% at days 5 and 10 compared to samples at day 0. Thereafter, cell migration of samples at day 20 returned to the value registered for samples at day 0 (Figures 4 and 5).

Meanwhile, samples stored in 90% O_2_ + 10% CO_2_ at day 10 showed a cell migration 30% higher than samples at day 0; then, cell migration decreased and it was equal to samples at day 0 at the end of storage (day 20) (Figures 4 and 5). Latter results suggest a potential enhanced wound healing capacity of strawberry bioactive compounds in response to the abiotic stress conditions faced during the refrigerated storage.

Andean blackberry and Andean blueberry fruits attenuated the oxidative damage elicited in human dermal HDFa fibroblasts by the stressor 2,2’‐azobis(2‐amidinopropane) dihydrochloride, suggesting these results promising benefits for the prevention of oxidative damage and skin injury (Alarcón‐Barrera et al., 2018). Moreover, the improvement in the fibroblast functionality and, in turn, in the cell migration exerted by extracts prepared from berries such as strawberry and blackberry (Van de Velde et al., 2018) and from black soybean seed coasts (Nizamutdinova et al., 2009) were associated with the phenolic compounds, specifically with the anthocyanins. The mechanism by which anthocyanins may promote the wound healing process in fibroblasts and keratinocytes was related to an increase in the production of the wound‐induced vascular endothelial growth factor (VEGF) (Nizamutdinova et al., 2009). Moreover, anthocyanins also inhibited ROS production and VEGF synthesis in endothelial cells stimulated with TNF‐α, and reduced, in a dose‐dependent manner, the adhesion of inflammatory monocytes to endothelial cells (Nizamutdinova et al., 2009).

Therefore, as the additional potential in the migration of fibroblasts exerted by strawberry extracts seems to be associated with the anthocyanins, the increment of these bioactive compounds experimented by samples stored in 90% O_2_ + 10% CO_2_ between days 5 and 10, early demonstrated by Van de Velde et al., (2019), would explain the better migration results presented in this work for these samples in that period of time.

## 4 CONCLUSIONS

The storage of strawberries in 70% O_2_ + 20% CO_2_ maintained or slightly decreased the antioxidant, anti‐inflammatory, and wound healing properties of fruits until 20 days. However, the storage of strawberries in 90% O_2_ + 10% CO_2_ between days 5 and 10 of storage improved the antioxidant capacity, increased the reduction of the pro‐inflammatory genes Cox‐2 and iNOS, and promoted a higher fibroblast migration that supposed an improved wound healing effect. The postharvest storage of strawberries in the atmosphere 90% O_2_ + 10% CO_2_ can be a promising alternative to offer fruits with an enhanced bioactivity.

## CONFLICT OF INTEREST

The authors declare no conflict of interest.

## ETHICAL APPROVAL

All cell studies were conducted under the Biological Use Authorization, BUA (Pharmacological characterization of plants for anabolic and cosmetic applications; approved 3 December 2018) authorized by the ethical committee review board at North Carolina State University (Institutional Biosafety Committee, IBC).
